# High expression of the p53 isoform γ is associated with reduced progression-free survival in uterine serous carcinoma

**DOI:** 10.1186/s12885-018-4591-3

**Published:** 2018-06-25

**Authors:** Katharina Bischof, Stian Knappskog, Ingunn Stefansson, Emmet Martin McCormack, Jone Trovik, Henrica Maria Johanna Werner, Kathrine Woie, Bjorn Tore Gjertsen, Line Bjorge

**Affiliations:** 1 Centre for Cancer Biomarkers (CCBIO), Department of Clinical Science, Precision Oncology Research Group, University of Bergen, Bergen, Norway; 2 Department of Gynecology and Obstetrics, Haukeland University Hospital, N-5021 Bergen, Norway; 3 Department of Oncology, Haukeland University Hospital, 5021 Bergen, Norway; 4 Section of Oncology, Department of Clinical Science, University of Bergen, Bergen, Norway; 5 Department of Pathology, Haukeland University Hospital, 5021 Bergen, Norway; 6 CCBIO, Department of Clinical Medicine, Section for Pathology, University of Bergen, Bergen, Norway; 7 Bergen Gynaecologic Cancer Research Group, Department of Clinical Medicine, University of Bergen, Bergen, Norway; 8 Department of Internal Medicine, Haematology Section, Haukeland University Hospital, 5021 Bergen, Norway

**Keywords:** Uterine serous carcinoma, Type II endometrial cancer, p53 isoforms, RT-qPCR, mRNA expression analysis, Biomarker

## Abstract

**Background:**

Uterine serous carcinoma (USC) is a rare but aggressive subtype of endometrial carcinoma. Large-scale comprehensive efforts have resulted in an improved molecular understanding of its pathogenesis, and the p53 pathway has been proposed as a key player and is potentially targetable. Here we attempt to further portray the p53 pathway in USC by assessing p53 isoform expression.

**Methods:**

We applied quantitative Real-Time PCRs (RT-qPCR) for expression analyses of total p53 mRNA as well as quantitative distinction of p53β, p53γ, and the total mRNA of amino-terminal truncated Δ40p53 and Δ133p53 in a retrospective cohort of 37 patients with USC. *TP53* mutation status was assessed by targeted massive parallel sequencing. Findings were correlated with clinical data.

**Results:**

The p53 isoform expression landscape in USCs was heterogeneous and dominated by total Δ133p53, while the distinct p53β and p53γ variants were found at much lower levels. The isoform expression profiles varied between samples, while their expression was independent of *TP53* mutation status. We found high relative p53γ expression to be associated with reduced progression-free survival (PFS).

**Conclusions:**

This is the first indication that elevated p53γ expression is associated with reduced PFS in USC. This single-center study may offer some insight in the landscape of p53 isoform expression in USC, but further validation studies are crucial to understand the context-dependent and tissue-specific role of the p53 isoform network in gynecological cancer.

**Electronic supplementary material:**

The online version of this article (10.1186/s12885-018-4591-3) contains supplementary material, which is available to authorized users.

## Background

Endometrial cancer is the most common gynecological malignancy in the Western world [1, 2], with the incidence increasing in recent years [3]. Traditionally, endometrial neoplasms are categorized into Type I and Type II cancers [4]. While the majority of tumors are classified as Type I, which are usually highly differentiated and thus less aggressive, around 20% of patients suffer from clinically aggressive Type II cancers that in a much higher percentage have extrauterine spread at the time of diagnosis and account for the majority of cancer related deaths [4–6].

Through large-scale comprehensive molecular characterization, endometrial carcinomas have recently been classified into four specific subgroups and we have gained a new understanding of the biology of USC [7]. The serous subtype has been shown to molecularly resemble basal-like breast cancers as well as high-grade serous ovarian carcinomas [7]. In USC, somatic mutations in the *TP53* gene are a common characteristic and seen in more than 90% of cases, resulting in genetic instability and widespread copy-number alterations. Although other subtype-specific molecular features are present, including increased transcriptional activity of genes such as *CCNE1* or *MYC*, that are involved in cell cycle regulation [7], it has become increasingly clear that the p53 pathway may be a key player in the genesis of USCs [8].

Mutant p53 proteins have been shown not only to lose their tumor-suppressive functions but also to gain oncogenic traits [9] Additionally, p53 function has recently been shown to be modulated by a number of alternative mechanisms through a network of structurally similar proteins in the p53 pathway [10, 11]. In *TP53* wild-type USC, inactivation of the p53 pathway must occur through alternative cellular mechanisms.

High-throughput RNA sequencing has produced vast amounts of data showing that more than 90% of human protein-coding genes produce multiple mRNA isoforms through such posttranscriptional mechanisms as non-canonical splicing and the use of alternative promoters [12, 13]. In humans, at least 12 different protein-encoding transcripts from the *TP53* locus have been reported; these are suggested to contain detectable, predictive, and prognostic markers to guide patient treatment in a large number of cancers [14]. The complex isoform composition limits the ability to quantitatively distinguish the amino-terminally truncated variants Δ40p53 and Δ133p53 into α, β, and γ. For the carboxy-terminal (C-terminal) altered variants p53β and p53γ and the amino-terminally (N-terminally) truncated isoforms Δ40p53 and Δ133p53, mRNA expression levels have been shown to be associated with tumor characteristics and aggressiveness in other p53*-*disrupted malignancies, such as breast cancer and epithelial ovarian carcinoma among others [11, 15–23].

In acute myeloid leukemia (AML), *TP53* mutation represents a rare event. In this context, p53β and p53γ protein isoforms have been shown to positively correlate with the *NPM1* mutation marker for survival, overall survival and response to chemotherapy. [24] Thus, the highly abrogated p53 pathway may represent an attractive future therapeutic target [25, 26].

**Fig. 1:**
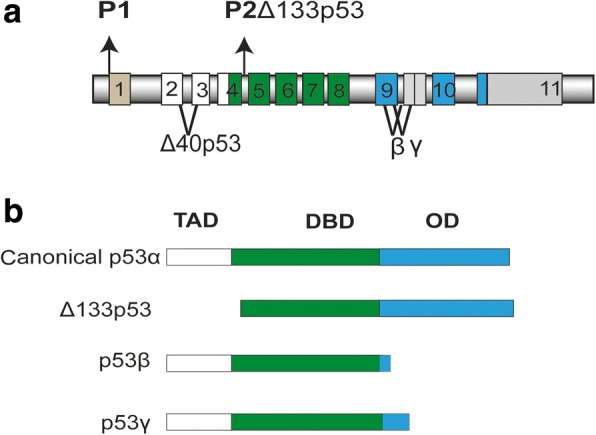
The p53 protein. **a** Structure of the human TP53 gene comprising 11 exons. P1 = proximal promoter encoding full-length p53, P2 = internal promoter resulting in Δ133p53 product. Alternative splicing sites (^)**. b** Illustrates the exon composition of relevant p53 isoforms. Abbrevations: Transactivation domain (TAD), DNA binding domain (DBD), C-terminal oligomerization domain (OD)

While the functional roles of p53 isoforms have been studied in the past both in vitro and in a number of clinical studies, to the best of our knowledge, a characterization of p53 isoform expression in USC tissue has never been reported. In this study, we present the mRNA expression analysis of the main p53 isoforms (the exon composition of relevant p53 isoforms is illustrated in Fig. 1b) in combination with *TP53* mutational status in serous endometrial cancers. Although the field of p53 research is constantly producing new insights into p53 structure and function, we are the first to offer an overview of the p53 isoform expression profiles of the main p53 isoforms in combination with *TP53* mutational status in serous endometrial cancers.

## Methods

### Patient characteristics and tumor specimens

Between June 2001 and April 2013, a total of 79 women were diagnosed with and treated for USC at Haukeland University Hospital, Bergen, Norway. For 37 of the patients, biological material for DNA sequencing and mRNA expression analysis was available together with prospectively collected clinicopathological data. The following parameters from our clinical database were relevant in this study: age at primary treatment, FIGO 2009 stage, level of complete cytoreduction and progression-free survival (PFS). PFS was defined as time in months from the last day of primary treatment to disease recurrence defined by RECIST criteria [27] Women were followed for a mean of 35 months (range 2–113), with the last follow-up entry in April 2015.

Tumor samples were acquired from hysterectomy specimens or diagnostic biopsies and included in the Bergen Gynecologic Cancer Biobank (REK Vest: Reference ID: 2014/1907). After collection at the time of primary diagnosis, tumor tissue was immediately frozen in liquid nitrogen. The tumor content of fresh frozen specimens was assessed in ethanol-fixed and hematoxylin- and eosin-stained sections. While the minimum cutoff for inclusion was set to 50%, tumor purity was above 80% in the majority of tissue samples studied. The histopathological analysis was performed at the Haukeland University Hospital, Department of Pathology. Specimens were fixed in buffered formaldehyde, embedded in paraffin and further processed in the laboratory before standard histological sections were made. Trained gynecologic pathologists performed the diagnostic assessments. This material has been reviewed previously [28].

### Nucleic acid isolation and cDNA synthesis

DNA was isolated by tissue digestion overnight at 65°C in lysis buffer containing NaCl, EDTA 0.5 M pH 8.5, TrisM pH 8, sodium dodecyl sulfate (SDS) 5%, proteinase K 20 mg/ml and H_2_O, followed by standard ethanol precipitation with sodium perchlorate and isopropanol. DNA quantity was determined using a Qubit Fluorometer (Thermo Fisher Scientific, Waltham, MA, United States of America). RNA was extracted using the RNeasy Mini Kit (Qiagen, Hilden, Germany) according to the manufacturer’s instructions and quantified using a NanoDrop M-1000 Spectrophotometer (Thermo Fisher Scientific, Waltham, MA, United States of America) and Agilent 2100 Bioanalyzer (Agilent Technologies, Santa Clara, CA, United States of America). Single-strand cDNA was synthesized from 500 ng total RNA in a 20μL reaction mix, using the Transcriptor reverse transcriptase system (Roche, Basel, Switzerland) according to the manufacturer’s protocol.

### Quantitative real-time PCR

Quantitative PCRs (qPCR) were performed using specific primers and hydrolysis probes targeting *TP53* on a LightCycler 480 instrument (Roche, Basel, Switzerland). Reaction mixes were prepared using the LightCycler 480 ProbesMaster kit (Roche, Basel, Switzerland), according to the manufacturer’s instructions. Relative mRNA expression levels of the p53 transcripts were normalized to an internal reference, *RPLP2* gene expression, amplified together with the p53 amplifications in a two-color duplex reaction. Primers/probes for detection of total p53, and mRNAs with the characteristic breakpoints for p53β, p53γ, Δ40p53 and Δ133p53, as well as *RPLP2* are listed in Additional file 1: Table S1; they were designed to be used under the same conditions in the qPCR amplification. All individual assays were validated on control DNA (plasmids) containing the specific isoform breakpoints and tested for cross-reactions against the other isoforms. Given this design, our assays for Δ40p53, Δ133p53 yielded data representing the total pool of molecules harboring these breakpoints, including both the canonical p53α and the p53β and p53γ forms in the exon 9–10 region. Our p53β and p53γ assays were specific for these breakpoints and included the different N-terminal isoforms. Thermocycling conditions for the qPCR were an initial denaturation step at 95 °C for 5 min, followed by 50 cycles of denaturation for 10 s at 95 °C and annealing / elongation at 53 °C for 20 s. Comparison between samples was performed using the ΔΔCt-method. Each analysis was performed in triplicate.

### *TP53* mutation calling

*TP53* mutation status was extracted from targeted massive parallel sequencing of tumor DNA. A total of 1000 ng of dsDNA was fragmented using the Covaris® M220 Focused-ultrasonicator™ (Covaris, Woburn, MA, United States of America). Library preparation was performed using the Agilent SureSelect XT-kit (Agilent Technologies, Santa Clara, CA, United States of America). All samples were run on a MiSeq instrument (Illumina, San Diego, CA, United States of America), and preliminary mutation calling was performed using the MiSeq reporter (MSR) software. From the raw mutation calling output, post-processing filters were applied and all suspected *TP53* mutations were validated by manual inspection of sequencing reads using the Integrative Genomics Viewer [29].

### Statistical analyses

The Shapiro-Wilk test was applied to assess the normality assumption. As the distribution of total p53, Δ40p53, Δ133p53, p53β and p53γ was non-Gaussian, Spearman correlation was calculated for continuous variables and Mann-Whitney U test was used to identify correlations between continuous data (age, PFS, FIGO stage and isoform expression levels) and *TP53* mutation status, presence of complete cytoreduction or age grouped by median. Fisher’s exact test was used for comparisons of categorical variables (patient age, presence of complete cytoreduction and mutation status). Multiple linear regression was used to assess whether confounding was present. Survival analyses were performed by the Kaplan-Meier method, and subsets of patients (divided by median relative expression of isoforms) were compared using the log-rank test. All *p*-values are two sided and *p*-values < 0.05 were considered significant. Statistical analyses were performed using the software package SPSS 22.0 (SPSS Inc., Chicago, IL, United States of America).

## Results

### Patient characteristics

A total of 37 patients diagnosed with USC were included. The mean and mode age at time of diagnosis was 74 and 73 years, respectively (range 56–88 years). The mean PFS was 14 months (range 0–96 months). A substantial number of patients were diagnosed in the early stages of the disease as 41% (15 of 37) and 8% (3 of 37) of the women presented with FIGO stage I and stage II, respectively. In 32% (12 of 37) of cases, the tumors were classified as stage III, while stage IV disease was diagnosed in 19% (7 of 37) of cases. Primary debulking surgery was performed in all but one woman, who was regarded as inoperable due to advanced age, reduced performance status and advanced disease. Complete cytoreduction was achieved in 72% (26 of 36) of the women undergoing surgery, while optimal debulking could not be accomplished in 28% (10 of 36) of cases. 62% (23 of 37) of patients were additionally treated with adjuvant platinum-containing chemotherapy (Table 1).

**Table 1:** Clinical-pathological characteristics of USC patients (*n* = 37)

Clinical parameters	
	mean (range)
Age at diagnosis; in years	74 (56–88)
Follow-up time; in months	35 (2–113)
Progression-free interval; in months	14 (0–96)
	N (%)
FIGO 2009 stage	
IA	5 (14%)
IB	10 (27%)
II	3 (8%)
IIIA	2 (5%)
IIIC	10 (27%)
IVA	1 (3%)
IVB	6 (16%)
Level of surgical cytoreduction ^a^	
Complete	26 (72%)
Residual disease	10 (28%)
Adjuvant chemotherapy	
Yes	23 (62%)
No	14 (38%)

^a^Primary debulking surgery was performed on 36 out of 37 women

### mRNA expression patterns of p53 isoforms in USC

We assessed the expression levels of total p53 mRNA, the C-terminal truncated isoforms p53β and p53γ, and the N-terminal truncated variants Δ40p53 and Δ133p53 by Real-Time qPCR. Notably, our assays were splice site specific, as such, combinations of alternative variants in the C- and N-terminal (variants with multiple alternative splice sites) were not discriminated. The Δ133p53 variant was detected alone or in combination with other p53 isoforms in 97% (36/37) of samples. The carboxy-terminal isoforms p53β and p53γ could be identified in 78% (29/37) and 76% (28/37) of specimens, respectively. In our dataset, all but two samples that expressed p53γ also expressed p53β. Moreover, in 25 of the samples in which p53β was detectable, p53γ was also expressed. A total of 70% (26/37) of samples expressed the combination of p53β and p53γ as well as Δ133p53 mRNA. We were not able to detect expression of Δ40p53 mRNA in any cases.(for illustration see Table 2).

**Table 2:** Overview over relative expression of p53 isoforms in individual patients together with progression free-survival

Patient ID	Δ133p53ratio	p53βratio	p53γratio	Progression free- survival (months)
1	67.16	6.05	.86	29
2	19.17	4.90	0	0
3	187.50	17.35	0	0
4	19.87	0	0	14
5	0	0	0	7
6	17.21	0	0	1
7	42.31	17.08	27.85	3
8	234.46	0	0	14
9	22.37	0	2.63	10
10	5.16	.79	.57	5
11	9.47	6.35	0	58
12	7.93	1.68	.29	96
13	2.81	.27	.45	25
14	2.65	.67	.66	11
15	4.60	3.23	1.02	11
16	6.78	3.60	1.35	2
17	2.26	1.30	.74	0
18	5.75	2.10	.93	2
19	10.55	3.09	1.56	19
20	28.51	1.48	2.14	0
21	28.63	18.59	7.90	1
22	19.20	2.52	.54	31
23	35.80	18.67	5.20	15
24	33.33	2.89	.34	43
25	36.17	4.86	4.62	0
26	97.55	60.37	3.39	0
27	10.57	5.58	1.44	1
28	6.16	.98	.48	4
29	42.70	8.18	2.23	48
30	10.28	4.42	.61	28
31	60.74	0	0	11
32	38.58	5.87	2.71	0
33	10.13	2.66	2.23	0
34	85.68	0	0	11
35	7.51	0	1.94	15
36	25.74	7.85	1.64	6
37	32.86	13.85	6.09	0

**Fig. 2:**
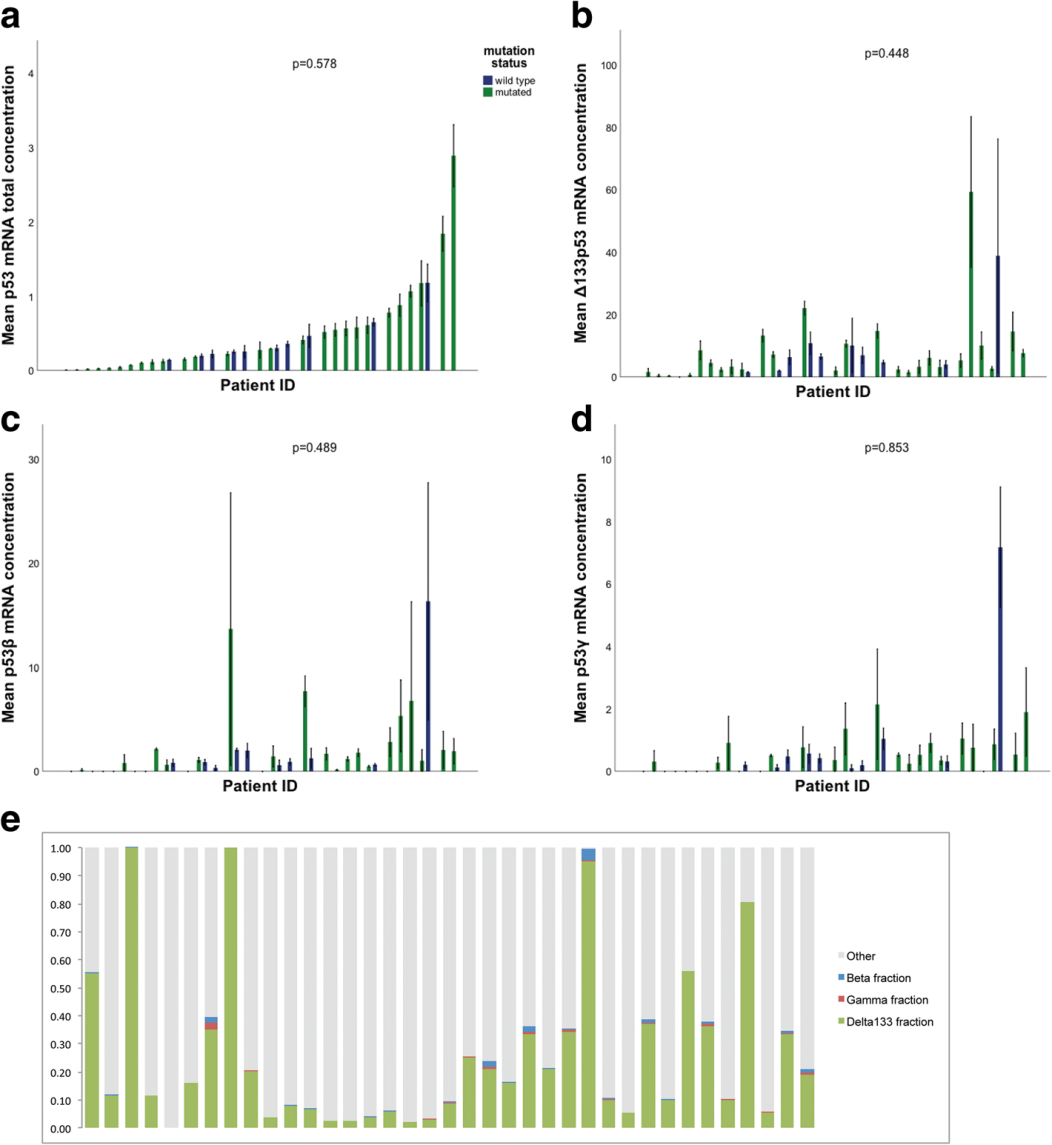
mRNA expression levels of p53 isoforms in individual USC tumors. The green bars represent *TP53* mutated specimens and the blue bars represent *TP53* wild-type tumors. Non-significant differential distribution of mRNA expression, error bars represent +/− 1 standard deviation in (**a**) total p53, cases were plotted in an ascending fashion. This order was maintained in plot **b**, **c** and **d**. **b** Δ133p53 (**c**) p53β and (**d**) p53γ in mutated versus wild-type tumors. **e** Histogram displaying fractions of p53 isoforms to total p53 mRNA in individual specimens

The p53 isoform expression levels were found to vary considerably from patient to patient (Fig. 2a–d). The largest variability was observed for Δ133p53, where we detected a 149-fold difference between the highest and the lowest expressing samples. The p53β and p53γ isoforms showed a variability of 109-fold and 70-fold, respectively. While Δ133p53 consistently accounted for the majority of isoforms expressed, p53β constituted a maximum 4% of isoforms expressed, while p53γ only was detectable in very low concentrations (Fig. 2e).

**Fig. 3:**
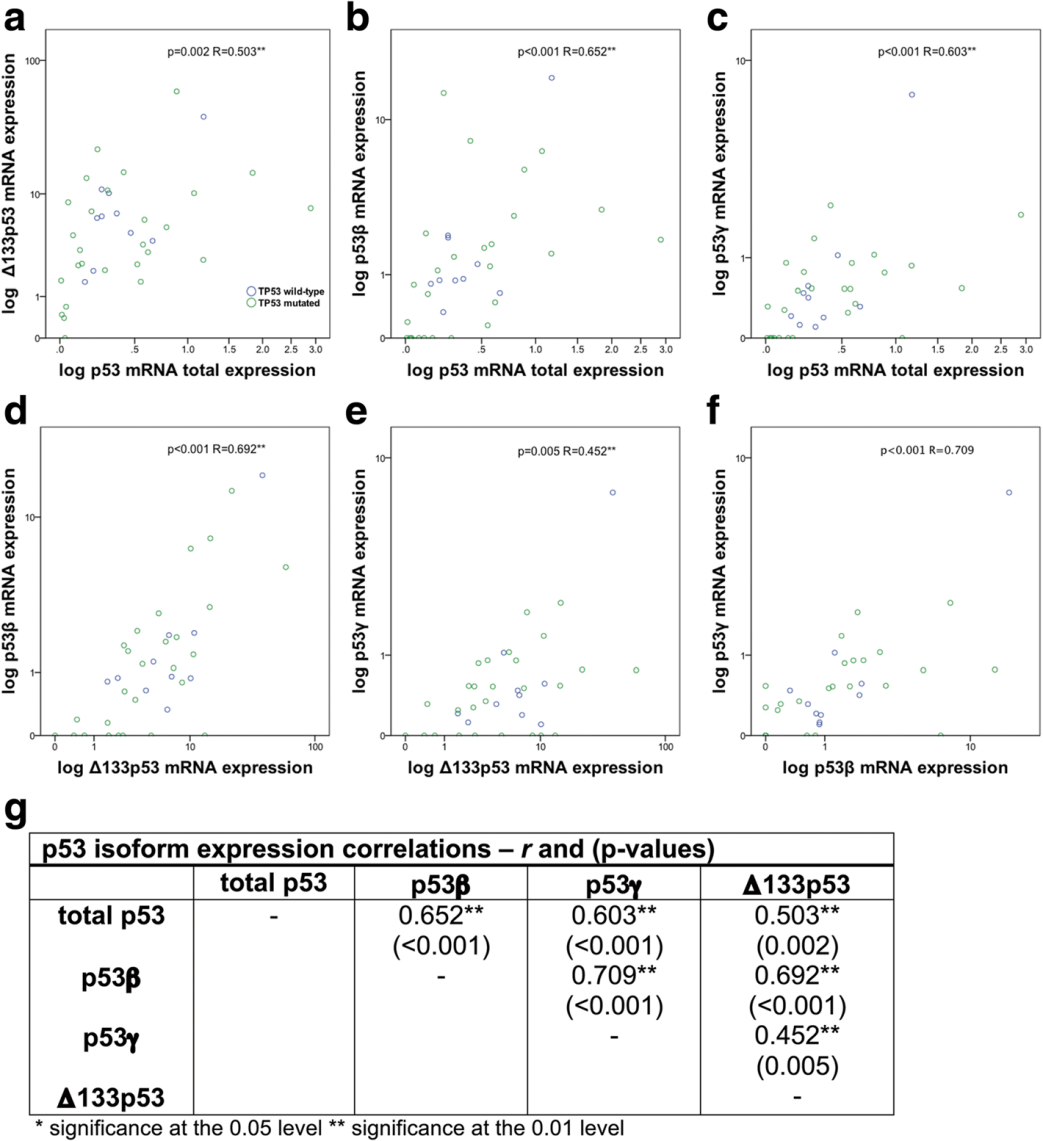
Pair by pair scatter plots demonstrating mRNA expression levels in tumors. Tp53 mutated specimens are depicted in green color, while TP53 wild-type tumors are shown as blue dots. Expression of total p53 versus levels of (**a**) Δ133p53 (**b**) p53β (**c**) p53γ as well as expression of p53 isoforms among each other (**d**) Δ133p53 to p53β (**e**) Δ133p53 to p53γ (**f**) p53β to p53γ. **g** Spearman correlation coefficients and *p*-values for univariate correlation of expression of total levels of p53 and levels of the individual p53 isoforms

The mRNA expression levels of the p53β, p53γ, and Δ133p53 isoforms were all significantly associated with total p53 expression levels and each other (Fig. 3). We found that the total p53 expression levels correlated with the levels of Δ133p53 (*R* = 0.503, *p* = 0.002), p53β (*R* = 0.652, *p* < 0.001) and p53γ (*R* = 0.603, *p* < 0.001) (Fig. 3a–c). In addition, the relative expression levels of p53β, p53γ and Δ133p53 isoforms within the tumors were highly correlated with one another. Expression of Δ133p53 was significantly associated with p53β (*R* = 0.692, *p* < 0.001) and p53γ (*R* = 0.452, *p* = 0.005). The p53β expression was linked with p53γ mRNA levels (*R* = 0.709, *p* < 0.001) (Fig. 3d–f).

### *TP53* mutation status in USC

In order to stratify p53 isoform expression data for *TP53* mutational status, targeted sequencing was performed. We detected somatic mutations in the *TP53* gene in 27 out of 37 (73%) tumor samples (Table 3).

**Table 3:** Overview over *TP53* mutations observed in patients

Patient ID	FIGO 2009 tumor stage	Mutation^a^	Amino acid change	Effect	Progression free survival (months)
1	1c	7577120C > T	R273H	missense^c^	29
2	4b	7577097C > T	D281N	missense^b^	0
3	4b	7577538C > T	R248Q	missense^c^	0
4	1b	7577581A > G	Y234H	missense^b^	14
5	3c	7,578,394 T > A	H179L	missense^b^	7
6	2b	7577539G > A	R248W	missense^c^	1
7	3c	7578239C > A	E204 ^a^	nonsense^b^	3
8	1c	7577120C > T	R273H	missense^c^	14
9	1a	7577573G > T	Y236 ^a^	nonsense^b^	10
		7578211C > A	R213L	missense^b^	
10	3a	7,577,580 T > C	Y234C	missense^b^	5
11	4b	7577539G > A	R248W	missense^c^	58
12	1c	7577153C > A	G262 V	missense^b^	96
13	3c	7577538C > T	R248Q	missense^c^	25
14	3c	7578508C > T	C141Y	missense^b^	11
15	1c	7,578,442 T > C	Y163C	missense^b^	11
16	3c	7578406C > T	R175H	missense^c^	2
17	4b	7577586A > T	I232N	missense^b^	0
18	3c	7577538C > T	R248Q	missense^c^	2
19	1a	7578406C > T	R175H	missense^c^	19
21	2a	7579556ATCAA		frameshift	1
23	1b	7577535C > G	R249T	missense^b^	15
25	4a	7578272G > A	H193Y	missense^b^	0
26	3c	7577121G > A	R273C	missense^b^	0
31	3c	7577569TGT		non-frameshift deletion	11
32	3c	7,578,190 T > C	Y220C	missense^b^	0
34	1a	7577141C > T	G266E	missense^b^	11
35	3a	7577130A > G	F270 L	missense^b^	15
		7577132C > T	S269 N	partially functional^b^	

^a^Coordinates; GRCh37

^b^Mutation has been reported earlier in the IARC archive [33]

^c^ Hot-spot mutation region

Notably, we found two of the samples to harbor two mutations. The most frequent point mutation detected was R248Q, observed in three patients. Several other hot-spot mutations such as R175H and R273H, were observed. Total p53 expression levels (*p* = 0.578) as well as the splice variants Δ133p53 (*p* = 0.448), p53β (*p* = 0.489) and p53γ (*p* = 0.853) were all independent of *TP53* mutation status (Fig. 2a–d).

### Prediction of clinical features by p53 isoform expression

**Fig. 4:**
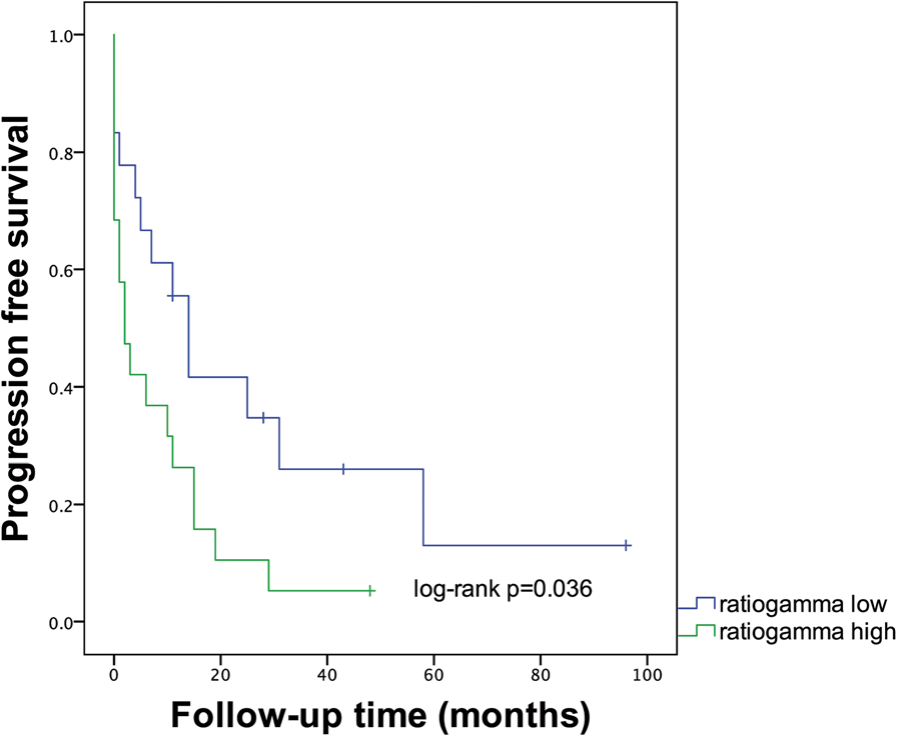
Differential progression free survival in patients. Patients are grouped by median as expressing high p53γ relative to total p53 (ratiogammahigh) versus low p53γ relative to total p53 (ratiogammalow)

We tested whether the expression patterns of Δ133p53, p53β and p53γ correlate with disease characteristics and identified a significant association between patient age and concentration of total p53 (*p* = 0.031). However, age was no longer significantly associated with expression of total p53 (*p* = 0.295) for multiple linear regression that introduced expression of the p53β, p53γ, and Δ133p53 isoforms as covariates. For the other p53 splice variants, no relationship was established between mRNA expression levels and age or relevant tumor traits, such as stage at primary diagnosis and tumor resectability. Relative expression levels of the p53γ isoform had an impact on time to relapse after primary treatment was completed. Our data showed that higher ratios of p53γ to total p53 were associated with shorter PFS (log-rank *p* = 0.036) as illustrated in Fig. 4. No such correlation was found for the Δ133p53 and p53β isoforms.

### *Tp53* mutation status and clinical parameters

Both the patient characteristics, such as age (*p* = 0.180) and the tumor features, such as FIGO stage at primary diagnosis (*p* = 0.271) and presence of complete cytoreduction in operated patients (*p* = 0.580), were independent of *TP53* mutation status. We found the mean PFS in patients with *TP53* wild-type cancers to be 18 months versus 15 months among women with tumors harboring mutated *TP53*. However, this difference was not statistically significant (log-rank *p* = 0.399).

## Discussion

Over the last decade, the description of splice variants of the *TP53* gene has dynamically reformed the p53 field, and p53 isoforms have emerged as possible active contributors in cancer formation and progression [30] We have previously described that p53β and p53γ protein expression correlates positively with overall survival, chemotherapy response and mutational markers for survival in the aggressive blood cancer AML [24] Leukemia in general has a low occurrence of *TP53* mutations. It was therefore interesting to examine whether the expression levels of p53β, p53γ, or other isoforms had a prognostic implication in tumors with a high frequency of *TP53* mutations. A recent publication by Shen et al. showed not only that mRNA isoform variations are associated with clinical outcomes in TCGA breast cancer data, but also that alternative splicing-based survival predictors consistently outperform gene expression-based prognosticators [31].

The focus of the present study was to assess the role of p53 splice variants in USC in a well-defined cohort of patients by performing highly sensitive quantitative Real-Time PCR. We detected expression of p53 isoforms in 97% of cases and found that expression levels varied considerably from patient to patient. Specifically, we found that the expression levels of the p53 isoforms p53β, p53γ, and Δ133p53 were all associated with total p53 expression levels. Whether this indicates that isoform expression is merely a side product of general *TP53* transcriptional activity in many patients remains unknown. Furthermore we show that the relative expression of p53β, p53γ, and Δ133p53 isoforms within the tumors were highly correlated with one another, consistent with findings in breast cancer [16].

The total Δ133p53 levels constituted the majority of p53 isoforms expressed in USC, while the levels of p53β and p53γ were much lower. The differential expression of Δ133p53 in cancerous cells seems to be highly dependent on the originating tissue. While an overexpression of Δ133p53 is seen in gastrointestinal tumors compared to cancer precursors [11] or paracancerous tissue [23], Van den Berg et al. showed that Δ133p53 variants were downregulated in the early stages of clear cell renal carcinoma [22]. There are conflicting reports regarding the clinical impact of Δ133p53 expression. The Δ133p53 β-variant has recently been linked to increased tumor invasiveness and worse prognosis in a cohort of breast cancers [17], but higher Δ133p53 levels have also been linked to favorable prognosis in *TP53* mutant advanced ovarian cancer [19] No such clinical associations of Δ133p53 expression were observed in our cohort.

We did not detect expression of the Δ40p53 isoform in any of the patients. This finding contrasts with several other forms of cancer. In breast cancer, Δ40p53 has been found to be the main isoform expressed, and it is significantly upregulated when compared to benign breast tissues, particularly in triple negative breast tumors [16] Hofstetter et al. showed the same significant upregulation of Δ40p53 in mucinous ovarian carcinomas in contrast to normal ovarian tissues and also indicated that higher expression of Δ40p53 constituted an independent prognosticator for longer PFS [20] In renal cell carcinoma, Δ40p53 was also present and significantly upregulated in the advanced stages [22].

For the p53β isoform, Avery-Kiejda et al. [16] reported an association between lower expression levels and diminished metastasis-free survival and a significant negative association with tumor size in a series of breast cancers. In our data, no such effect was seen.

Although the number of patients included in our study is limited, our data strongly suggest that high relative expression of p53γ is associated with shorter PFS. These findings are in contrast to data from breast cancer patients, where expression of p53γ has been linked to good prognosis in *TP53* mutant tumors [15] and to tumor grade in unselected breast cancers [16]. In vitro, p53γ has been shown to affect FLp53-dependent transactivation of Bax and is therefore believed to exert tumor-suppressive functions [10]. Furthermore, stable transfection of lung carcinoma cell lines with the p53β and p53γ isoforms has been shown to exert chemosensitizing effects. Unexpectedly, the same cells showed accelerated tumor growth when compared to null cells in an in vivo model [32]. Although p53γ was associated with shorter PFS and we observed a strong correlation between levels of p53γ and p53β, we could not establish an association between p53β and PFS.

Our data demonstrated that the *TP53* mutation rate was 73%, which is somewhat lower than in other studies [7]. This is probably due to random chance and the small cohort size. The number of patients with wild-type *TP53* was limited in our present study, but we found no indications that the expression levels of any of the three detected isoforms were associated with *TP53* mutation status in the tumors. This independence of isoform expression from *TP53* mutation status is in line with previous findings in ovarian carcinoma and breast cancer series [15, 17, 20]. In our correlation analysis, *TP53* mutation status could not predict cancer progression.

These data in combination highlight that p53 function is complex and must be regarded as a result of the precise and tissue-specific balance between expressions within the p53 isoform network [14]. It may well be, that the expression levels of certain isoforms may be associated with poor prognosis in some forms of cancer and good prognosis in others. Our data indicate that this may be the case for p53γ. We believe that our findings can help direct further study of p53 isoform expression in USC by introducing a potential, clinically applicable biomarker for future validation in other cohorts.

## Conclusions

The *TP53* mutational profile by itself appears not to contain any prognostic information for patients in this cohort. This single-center study may offer some insight in the landscape of p53 isoform expression in USC and introduces p53γ as a possible predictor of progression free- survival. The tissue-specific and complex regulation of the individual p53 isoforms must be understood before p53 isoforms can serve as predictive or prognostic biomarkers or therapeutic targets in USCs.

## Additional file

Additional file 1: **Table S1.** Primers and probes for qPCR. (DOCX 14 kb)

## Abbreviations

AML: Acute myeloid leukemia
Bax: Bcl-2-associated X protein
*CCNE1*: Cyklin E1 Gene
C-terminal: Carboxy-terminal
FIGO: The International Federation of Gynecology and Obstetrics
*MYC*: MYC proto-oncogene
N-terminal: Amino-terminal
PFS: Progression-free survival
RECIST: Response evaluation criteria in solid tumors
*RPLP2*: Ribosomal protein lateral stalk subunit P2
RT-qPCR: Quantitative real-time PCR
TCGA: The cancer genome atlas
*TP53*: Tumor protein p53
USC: Uterine serous carcinoma
