# Supplementary material for: High expression of the p53 isoform γ is associated with reduced progression-free survival in uterine serous carcinoma
Source: BMC Cancer. 2018 Jun 25;18:684. doi: 10.1186/s12885-018-4591-3 (PMC6019524; doi:10.1186/s12885-018-4591-3)
Supplement: Supplementary file 1 — Table S1. Primers and probes for qPCR. (DOCX 14 kb) [file 12885_2018_4591_MOESM1_ESM.docx]

**Table S1.** Primers and probes for qPCR

| **Primer / probe** | **Sequence** |
| --- | --- |
|  |  |
| *p53 total* |  |
| p53 total F | GAAGAGAATCTCCGCAAGAAAGG |
| p53 total R | TCCATCCAGTGGTTTCTTCTTTG |
| p53 total probe | 6FAM-AGCACTAAGCGAGCACTGCCCAACA-BBQ |
|  |  |
| *Δ40p53* |  |
| Δ40p53 | CAGACCTATGGAAACTGTGAGTGG |
| Δ40p53 | TCAGGAAGTCTGAAAGACAAGAGCAG |
| Δ40p53 | 6FAM-CCTAGCAGAGACCTGTGGGAAGCGAA-BBQ |
|  |  |
| *Δ133p53* |  |
| Δ133p53 F | ACTCTGTCTCCTTCCTCTTCCTACAG |
| Δ133p53 R | GTGTGGAATCAACCCACAGCT |
| Δ133p53 probe | 6FAM-TCCCCTGCCCTCAACAAGATGTTTTGCC-BBQ |
|  |  |
| *p53β* |  |
| p53β F | AACCACTGGATGGAGAATATTTCAC |
| p53β R | TCATAGAACCATTTTCATGCTCTCTT |
| p53β probe | 6FAM-CAGGACCAGACCAGCTTTCAAAAAGAAAATTGTT-BBQ |
|  |  |
| *p53δ* |  |
| p53δ F | AACCACTGGATGGAGAATATTTCAC |
| p53δ R | TCAACTTACGACGAGTTTATCAGGAA |
| p53δ probe | 6FAM-TTCAGATGCTACTTGACTTACGATGG-BBQ |
|  |  |
| *RPLP2* |  |
| RPLP2 F | gaccggctcaacaaggttat |
| RPLP2 R | ccccaccagcaggtacac |
| RPLP2 probe | Cy5-agctgaatggaaaaaacattgaagacgtc-BBQ |
